# Heparins that block VEGF-A-mediated von Willebrand factor fiber generation are potent inhibitors of hematogenous but not lymphatic metastasis

**DOI:** 10.18632/oncotarget.11832

**Published:** 2016-09-02

**Authors:** Lukas Goertz, Stefan Werner Schneider, Anna Desch, Frank Thomas Mayer, Julian Koett, Kai Nowak, Ioannis Karampinis, Michael K. Bohlmann, Viktor Umansky, Alexander Thomas Bauer

**Affiliations:** ^1^ Experimental Dermatology, Department of Dermatology, Venereology, and Allergy, Medical Faculty Mannheim, University of Heidelberg, Mannheim, Germany; ^2^ Department of Dermatology and Venereology, University Hospital Hamburg-Eppendorf, Hamburg, Germany; ^3^ Department of Surgery, Mannheim University Medical Center, Heidelberg University, Mannheim, Germany; ^4^ Department of Obstetrics and Gynaecology, Mannheim University Medical Center, Heidelberg University, Mannheim, Germany; ^5^ Skin Cancer Unit, German Cancer Research Center (DKFZ), Department of Dermatology, Venereology and Allergology, University Medical Center Mannheim, Ruprecht-Karl University of Heidelberg, Mannheim, Germany

**Keywords:** von Willebrand factor, metastasis, melanoma, angiogenesis, coagulation

## Abstract

Von Willebrand factor (VWF) serves as a nidus for platelet aggregation and thrombosis. We hypothesize that VWF fibers contribute to the development of venous thromboembolism (VTE) and to metastasis formation. Here, we show that vascular and lymphatic endothelial cells (ECs) express VWF *in vitro* and release VWF fibers after activation by tumor cell supernatants. In contrast, an *ex vivo* analysis of primary mouse tumors revealed the presence of VWF fibers in the blood microvasculature but not in lymphatic vessels. Unlike the anticoagulant Fondaparinux, an inhibitor of thrombin generation, the low-molecular-weight heparin (LMWH) Tinzaparin inhibited VWF fiber formation and vessel occlusion in tumor vessels by blocking thrombin-induced EC activation and vascular endothelial growth factor-A (VEGF-A)-mediated VWF release. Intradermal tumor cell inoculation in VWF- and ADAMTS13-deficient mice did not alter lymph node metastases compared with wild type animals. Interestingly, multiple tumor-free distal organs exhibited hallmarks of malignancy-related VTE, including luminal VWF fibers, platelet-rich thrombi and vessel occlusions. Furthermore, ADAMTS13 deficiency, characterized by prolonged intraluminal VWF network lifetimes, resulted in a severely increased number of metastatic foci in an experimental model of hematogenous lung seeding. Treatment with Tinzaparin inhibited tumor-induced release of VWF multimers, impeded platelet aggregation and decreased lung metastasis. Thus, our data strongly suggest a critical role of luminal VWF fibers in determining the occurrence of thrombosis and cancer metastasis. Moreover, the findings highlight LMWHs as therapeutic strategy to treat thrombotic complications while executing anti-metastatic activities.

## INTRODUCTION

Venous thromboembolism (VTE) is a life-threatening complication associated with a poor prognosis due to enhanced tumor progression in cancer patients [1, 2]. Among the different cancer types, the VTE incidence is especially high in malignant melanoma, occurring in approximately 25% of melanoma patients [3]. An association between an enhanced risk of thrombotic complications and an increase of von Willebrand factor (VWF) levels in plasma and tumor tissue of patients with distinct malignancies has been described by multiple clinical studies for specific malignancies [4–7]. VWF is a multimeric glycoprotein that has critical effects on hemostasis, angiogenesis, platelet recruitment [8] and thrombus formation [9]. Because it is generally accepted that platelets contribute to each step in metastasis formation [10–12], VWF may promote cancer dissemination and spread. Our own *in vitro* studies have revealed that melanoma cells activate vascular endothelial cells (ECs), thus resulting in the exocytosis of Weibel-Palade bodies (WPBs) and the release of procoagulatory, ultra-large von Willebrand factor (ULVWF) multimers from intact ECs [13–15]. Owing to high blood shear rates, these multimers unroll and assemble into VWF fibers that bind platelets and leukocytes, which mediate thrombotic and inflammatory responses, respectively [16–20]. Moreover, we have recently observed VWF fibers in the tumor vasculatures of melanoma patients and *ret* transgenic mice (a mouse model characterized by spontaneous melanoma development) [21]. Multiple mouse model- and human sample-based analyses have revealed that an increase in EC activation and a local inhibition of ADAMTS13 (**a d**isintegrin-like **a**nd **m**etalloproteinase with **t**hrombo**s**pondin type I repeats **13**), an enzyme that cleaves and inactivates VWF multimers under physiological conditions [22], are responsible for the occurrence of VWF fibers in the tumor microenvironment. Tinzaparin, a clinically approved low-molecular-weight heparin (LMWH), causes inhibition of EC activation and subsequent attenuation of VWF network formation and platelet aggregation, thus resulting in a tumor weight reduction and survival benefits [21]. Together with another study showing a lung metastasis reduction after antibody-mediated inhibition of VWF [23], our data support the hypothesis that VWF has pro-metastatic functions. In contrast Terraube et al. showed an increase of lung metastases in VWF-deficient mice pointing towards an anti-metastatic effect of VWF [24]. So far, no reliable data regarding the pro- or anti-metastatic properties of VWF are currently available. In this context, it should be emphasized that inducing lung metastasis through an intravenous infusion of tumor cells resembles the hematogenous route for metastasis formation, which is characterized by metastatic foci in lungs [25, 26]. However, tumor cells can disseminate via blood vessels and lymphatics, thus making evaluation of both pathways essential [27]. Although lymphatic ECs contain WPBs and store VWF [28], the roles of VWF in the lymphatics and in lymph node metastasis are unknown.

Here, we used *in vitro* settings and mouse models to examine malignant melanoma progression, and we used mice deficient in VWF (VWF −/−) and ADAMTS13 (A13 −/−) to evaluate whether VWF affects cancer-associated VTE and the formation of lymphatic and hematogenous metastases. Different anticoagulant heparins were screened for their capacities to block tumor-induced EC activation with subsequent ULVWF release, angiogenesis and metastasis.

## RESULTS

### Tumor-secreted VEGF-A activates VWF fiber formation in vascular and lymphatic ECs *in vitro*

To evaluate whether tumor cells stimulate the exocytosis of VWF in the lymphatic endothelium *in vitro*, we performed immunofluorescence studies and enzyme-linked immunosorbent assays (ELISAs). Since our previous studies have demonstrated that melanoma cells are able to activate vascular ECs [13, 14], human umbilical vein endothelial cells (HUVECs) were used as control.

**Figure 1:**
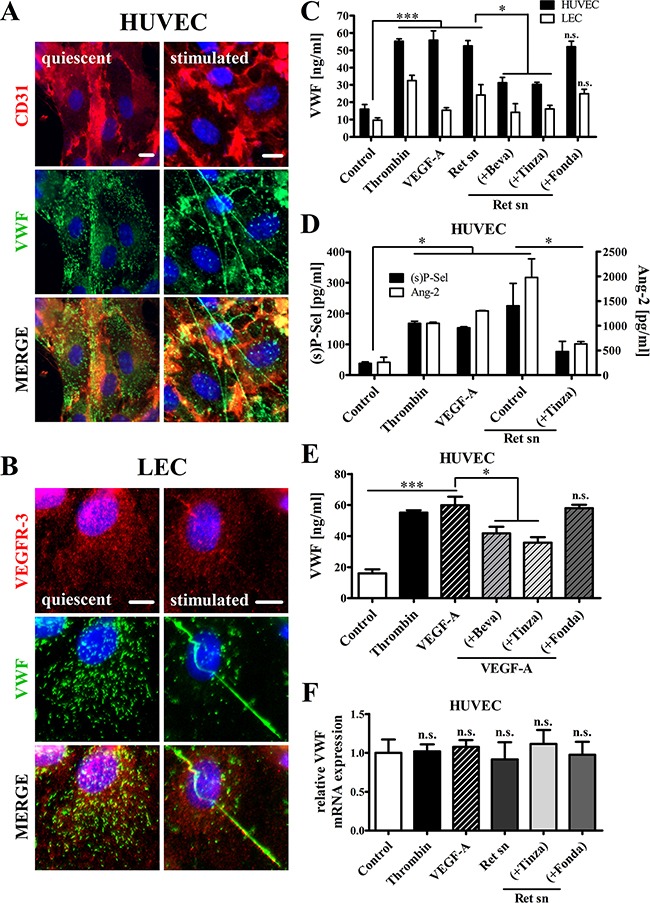
Tinzaparin, but not Fondaparinux, inhibits VEGF-A-mediated EC activation by Ret melanoma cells *in vitro* HUVECs or LECs were stimulated with VEGF-A or Ret supernatant (sn) alone or supplemented with Bevacizumab (+Beva), Tinzaparin (+Tinza) or Fondaparinux (+Fonda). Thrombin was used as positive control. **(A+B)** Immunofluorescence staining of VWF (green), CD31 (red; HUVECs), VEGFR-3 (red; LECs) and DAPI (blue) shows the intracellular localization of VWF in the quiescent endothelium. VWF release from stimulated endothelial cells is accompanied by the formation of VWF fibers. **(C)** ELISA shows a significant induction of endothelial VWF release by VEGF-A and Ret sn. **(D)**) VWF-release is accompanied by soluble P-selectin ((s)P-Sel) and angiopoietin-2 (Ang-2) release from activated HUVECs. **(E)** VEGF-A-mediated VWF release is attenuated by Bevacizumab and Tinzaparin but not by Fondaparinux. **(F)** RT-qPCR analysis was performed after stimulation of HUVECs with the indicated supplements for 24 hours. Data were normalized to Control. N = 4-12 from at least three independent experiments. Data are presented as the mean ± SEM. n.s. = not significant, *p < 0.05, **p < 0.01, ***p < 0.001 vs Control. Scale bars = 10 μm.

The immunofluorescence-based analyses demonstrated a characteristic punctuate, cytoplasmic VWF distribution in the quiescent endothelium, thus indicating that both HUVECs and lymphatic ECs (LECs) store VWF within WPBs *in vitro*. Tumor cell-induced stimulation of HUVECs and LECs triggered VWF release, evidenced by a reduction in stored VWF and the formation of VWF fibers on the intact endothelial surface (Figure 1A+1B). ELISA analysis indicated that Ret melanoma cell supernatants increased VWF exocytosis from HUVECs and LECs (Figure 1C). Notably, the tumor cell-induced release of VWF from HUVECs (52.6 ± 3.1 ng/ml) was twice that from LECs (24.3 ± 3.0 ng/ml). In addition, incubation of HUVECs and LECs with VEGF-A was sufficient to induce VWF secretion. In line with the results of previous studies [13, 21], the anti-VEGF-A-antibody Bevacizumab significantly reduced VEGF-A- and melanoma-mediated VWF release by approximately 40%, thus confirming the essential role of VEGF-A in EC activation. Next to Bevacizumab, the LMWH Tinzaparin also specifically blocked VWF secretion after incubation of LECs and HUVECs with supernatants of melanoma cells, whereas Fo ndaparinux showed no effect (Figure 1C). Significantly elevated levels of angiopoietin-2 and soluble P-selectin (stored in WPBs adjacent to VWF) confirmed the melanoma cell supernatant-induced activation of HUVECs and the ability of Tinzaparin to inhibit EC activation (Figure 1D).

We next examined the diverging functions of Tinzaparin and Fondaparinux on VEGF-A (Figure 1E). Reduced levels of VWF in the supernatant of a HUVEC monolayer confirmed the inhibition of VEGF-A by Tinzaparin. As expected, Fondaparinux showed no inhibition of VEGF-A-stimulated EC activation (Figure 1E). Since Tinzaparin exerts its activity by inhibiting thrombin generation and VEGF-A binding [21, 29], the anticoagulant activity of Fondaparinux is mediated solely through factor Xa inhibition and lacks the capacity to suppress VEGF-A [21] (Figure 1E). To exclude any contributions from endothelial VWF expression to these results, qRT-PCR was performed on RNA obtained from HUVECs after incubation with melanoma cell-derived supernatants for 24 h. The results demonstrated that VEGF-A, Tinzaparin and Fondaparinux did not have regulatory effects on VWF expression (Figure 1F). Thus, the distinct characteristics of these clinically approved anticoagulants make them ideal tools for analyzing the molecular mechanisms of tumor cell-induced EC activation.

### Tumor blood vessels exhibit luminal VWF fiber generation

**Figure 2:**
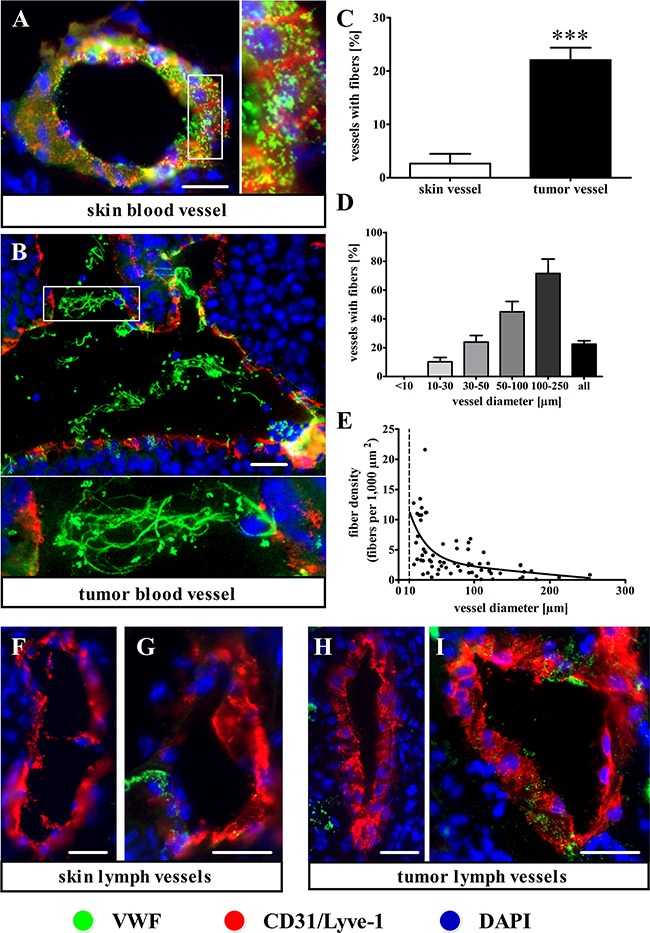
Immunofluorescence staining shows intraluminal VWF fiber formation in the tumor blood vessels of intradermal melanoma tissue **(A-E)** Cryosections of control skin and primary skin tumors were stained for VWF (green) and the endothelial cell marker CD31 (red). **(A)** In healthy skin vessels, VWF is stored in the endothelial cells of the vessel wall, indicating a quiescent endothelium. **(B)** Tumor vessels show intraluminal VWF fiber formation and reduced endothelial VWF in the blood vessel wall, indicating endothelial activation. **(C)** A quantitative analysis demonstrates an increased number of tumor vessels with VWF fiber formation, as compared with healthy skin control. **(D)** The percentage of tumor vessels with VWF fibers as a function of vessel diameter demonstrates that larger tumor vessels are more likely to contain luminal VWF fibers. There was no VWF fiber formation observed in vessels with diameters < 10 μm. **(E)** A fiber density analysis expressed as the number of fibers per 1,000 μm^2^ of luminal vessel area demonstrates an inverse correlation between the VWF fiber density and vessel diameter. **(F-I)** Immunofluorescence staining for the Lyve-1 lymphatic cell marker (red) shows that lymphatics rarely express VWF, with no VWF fiber formation in either the tumor or the healthy skin control. N = 76 – 313 vessels from 4 animals per group. Data are presented as the mean ± SEM. ***p < 0.001 vs the skin vessel. Scale bars = 20 μm.

To determine the *in vivo* relevance of VWF fibers in blood vessels, we analyzed primary skin tumors obtained from intradermal injections of Ret or B16F10 melanoma cells. To this end, tumor cryosections were stained for VWF and the endothelial marker CD31. Healthy skin as control showed a punctuate VWF distribution in the vessel wall without luminal VWF networks, indicative of a quiescent, inactive endothelium (Figure 2A). In contrast, the tumor microvasculature exhibited VWF fibers in the blood vessel lumina, correlating with a strong reduction in VWF stored in the endothelium. This indicates WPB exocytosis and luminal VWF release from the activated endothelium (Figure 2B). The corresponding quantification revealed that only 2.6 ± 1.8% of vessels in the healthy skin contained VWF fibers; this was significantly increased to 22.0 ± 2.3% in the Ret tumor tissue (Figure 2C, Supplementary Table S1) and to 27.6 ± 2.0% in B16F10 melanomas (Supplementary Figure S1; Supplementary Table S2). These VWF multimers are efficient binding partners for platelets mediating platelet aggregation and vessel occlusion (Supplementary Figure S2). A detailed analysis of the vasculature revealed a correlation between the presence of VWF fibers (defined as a fiber length > 5 μm) and the vessel diameter (Figure 2D). For example, 71 ± 10.1% of very large tumor vessels (> 100 μm), but only 10.1 ± 3.0% of small vessels (10 μm-30 μm), exhibited intraluminal VWF fibers. Notably, very small vessels (i.e., capillaries < 10 μm) did not show any fiber formation. Plotting the fiber density (fibers per 1,000 μm^2^ of luminal vessel area) as a function of vessel diameter (10 μm–250 μm) demonstrated that the fiber density was highest in small vessels and decreased with vessel size (Figure 2E). In summary, larger vessels are most likely to contain VWF fibers, but the fiber density becomes higher in smaller vessels once they are activated.

### Intratumoral lymphatic vessels lack luminal VWF fibers

We asked whether fiber generation could also occur in tumor lymphatics. To answer this question, we analyzed 62 lymphatic vessels in healthy skin as control and 80 intratumoral lymphatic vessels in primary Ret tumor cryosections stained for VWF and Lyve-1. In the healthy skin control group, only 1.6% of the lymphatic vessels exhibited VWF stored in the vessel wall (Figure 2F+2G). In the primary tumor tissues, 12.5% of all analyzed lymphatic vessels contained VWF, whereas the amount of VWF stored in the lymphatic endothelium was considerably lower than that in the blood vessels (Figure 2H+2I). Consequently, VWF fibers were undetectable within the lymphatic vessel lumina. In summary, VWF expression and intraluminal VWF fiber formation are scarcely present in healthy lymphatic vessels and tumor lymphatics.

### Tinzaparin inhibits VWF fiber generation in the tumor vasculature more efficiently than Fondaparinux

**Figure 3:**
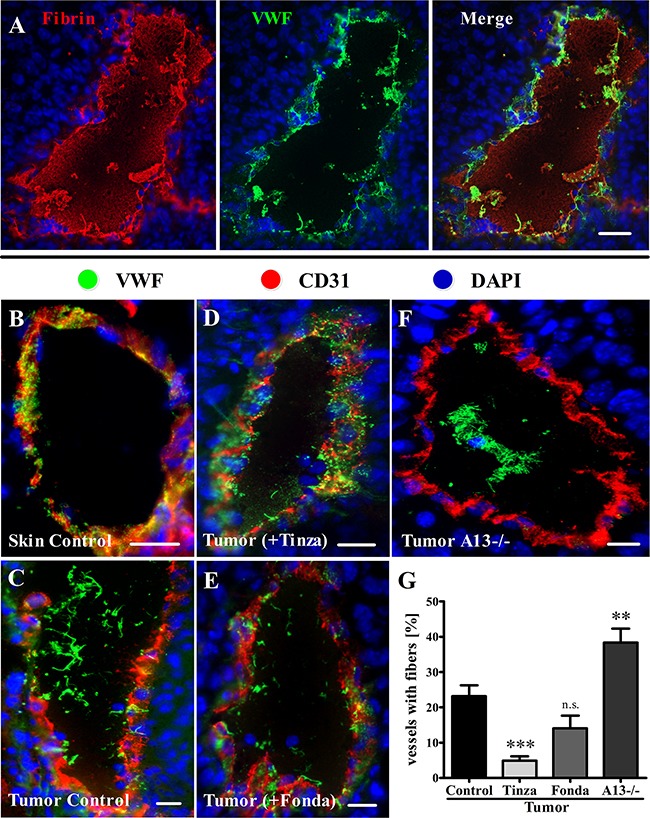
Tinzaparin reduces VWF fiber generation in tumor blood vessels more efficiently than Fondaparinux Immunofluorescence staining of cryosections from healthy skin or intradermally growing tumors was performed for VWF (green) and Fibrin (red) or CD31 (red). Nuclei were stained with DAPI (blue). **(A)** A representative image of a tumor blood vessel demonstrates intraluminal VWF fiber colocalization with fibrin fibers. **(B-F)** Representative tumor blood vessels stained for VWF and CD31. **(B)** Blood vessels in healthy skin show VWF storage in the vessel wall. **(C)** Tumor vessels in saline-treated control mice exhibit luminal VWF networks that correlate with reduced VWF levels in the vessel wall. **(D)** Tinzaparin (+Tinza) treatment correlates with a reduced level of VWF fibers in the vessel lumen and a punctate staining in the vessel wall. **(E)** Fondaparinux (+Fonda) inhibits EC activation with luminal VWF fiber formation to a lesser extent than Tinzaparin. **(F)** Tumor tissue from ADAMTS13-deficient animals (A13−/−) shows vessels with pronounced intraluminal VWF fibers. **(G)** A quantitative analysis reveals a significantly decreased number of vessels with intraluminal VWF with the Tinzaparin treatment compared to the control, whereas ADAMTS13 deficiency results in an increase in tumor blood vessels with VWF fibers. Tumor vessels (n = 1556 – 2369) from 7-14 animals per group were analyzed. Data are presented as the mean ± SEM. n.s. = not significant, **p < 0.01, ***p < 0.001 vs Tumor Control. Scale bars = 20 μm.

Fondaparinux and Tinzaparin exert their anticoagulant activities primarily by inhibiting thrombin generation [30]. However, unlike Fondaparinux, Tinzaparin exhibits a strong binding affinity for VEGF-A, thereby blocking its EC activation and angiogenesis activities [21]. The anticoagulant activities of these heparins were measured, and predictably, subcutaneous (s.c.) injections of Tinzaparin or Fondaparinux exhibited strong inhibitory effects on coagulation in a thrombin generation assay of mouse blood (Supplementary Figure S3). Once activated, thrombin drives the conversion of fibrinogen into fibrin [31]. Therefore, we used immunofluorescence microscopy to test the ability of the clinically approved anticoagulants to inhibit fibrin generation in the tumor vasculature (Supplementary Figure S4). As described above (Figure 2), the proportion of luminally released ULVWF in the tumor vasculature was strongly increased compared with that in control skin vessels. The VWF fibers colocalized with fibrin networks (Figure 3A). Compared with the vehicle-treated control, Tinzaparin and Fondaparinux resulted in a strong reduction or a total absence of intraluminal fibrin accumulation in tumor vessels. Quantification revealed a significant reduction of vessels with fibrin fibers to 24.3 ± 5.0% for Tinzaparin and to 23.8 ± 4.6% for Fondaparinux compared with 49.1 ± 3.4% for the untreated control (Supplemental Figure S4A-G; Supplementary Table S3), thus indicating strong anti-thrombin activities of both Tinzaparin and Fondaparinux. Because tumor cell-secreted VEGF-A is a mediator of EC activation in addition to thrombin [13, 14], VWF fibers were examined in tumor tissue. Treatment with Tinzaparin showed that VWF was primarily stored in the vessel walls, and few VWF fibers were detected in the vessel lumina (Figure 3D). This outcome resembled healthy skin vessels (Figure 3B) rather than the tumor vessels from vehicle-treated littermates (Figure 3C). Tinzaparin, which is a potent inhibitor of both thrombin formation and VEGF-A, significantly reduced the fraction of vessels with VWF networks to 4.9% ± 1.3% compared with 23.2% ± 3.1% in vehicle-treated control animals (Figure 3G; Supplementary Table S4). Importantly, inhibition of VWF fiber formation by Fondaparinux, a specific inhibitor of thrombin generation lacking a VEGF-A binding site, was not significant (14.1% ± 3.6%; Figure 3E+3G; Supplementary Table S4). To validate the specificity of these results, we evaluated intradermal tumors in VWF- and ADAMTS13-deficient mice. As expected, a VWF deficiency was characterized by a lack of VWF in the vessel wall and of the luminal fibers of the tumor vessels (data not shown). Additionally, the absence of ADAMTS13 correlated with prolonged ULVWF network lifetimes, reflected by a strong increase in tumor vessels containing VWF fiber formation in the tumor vasculature to 38.3 ± 3.9% (Figure 3F+3G; Supplementary Table S4). Together, the data suggest that inhibition of Xa-mediated thrombin generation by the pentasaccharide Fondaparinux is not sufficient to block tumor-associated EC activation and luminal VWF fiber formation in the microenvironment of the tumor. By contrast, inhibition of thrombin and VEGF-A by the LMWH Tinzaparin is required for an efficient blockage of EC activation and VWF fiber formation in the tumor vasculature. Therefore we conclude that VEGF-A and thrombin are redundant stimuli for tumor-associated EC activation. Our data show that both stimuli, i.e. with Tinzaparin, have to be blocked to prevent EC activation.

### Tinzaparin inhibits *in vitro* and *in vivo* angiogenesis

**Figure 4:**
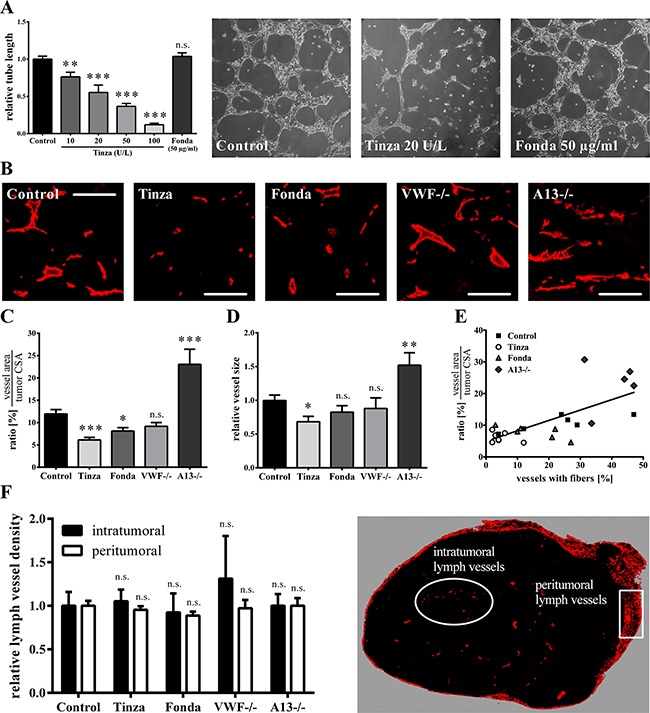
VWF fiber formation correlates with enhanced vascular angiogenesis **(A)** *In vitro* Matrigel angiogenesis assays were performed with HUVECs maintained in a culture medium supplemented with the saline control, Tinzaparin (Tinza) or Fondaparinux (Fonda). Tube length was quantified after 9 hours of incubation. This assay does not show an inhibitory effect of Fondaparinux on EC tube formation, but Tinzaparin reduces tube formation in a dose-dependent manner. Representative microscopic images show tube formation after 9h of incubation time with the control, Tinzaparin (20 U/L) or Fondaparinux (50 μg/ml). **(B-E)** Tumor angiogenesis in wild type mice (WT) treated with saline control, Tinzaparin or Fondaparinux and in VWF- (VWF−/−) and ADAMTS13-deficient (A13−/−) mice, analyzed via CD31 immunofluorescence staining in primary tumors. **(B)** Representative immunofluorescence images show vessel sizes and distributions in primary melanoma. **(C)**The percentage of the total vessel area on the tumor cross sectional area (CSA) shows that the Tinzaparin-mediated inhibition of angiogenesis is stronger than that of Fondaparinux. **(D)** Only Tinzaparin-treatment significantly reduces the average vessel size. **(E)** Vessel density as a function of the percentage of vessels with VWF fibers demonstrates a positive correlation between VWF fiber formation and vascular angiogenesis. **(F)** Primary tumors were stained for Lyve-1 to quantify lymphangiogenesis. To analyze the peritumoral lymphatic vessel density, the number of lymphatic vessels that crossed a 1-mm long line perpendicular to the primary tumor margin was determined. An analysis of intra- and peritumoral lymphatic vessel densities demonstrates that lymphangiogenesis is unaffected by endothelial cell activation and VWF fibers. N = 5 – 12 mice per group. Data are presented as the mean ± SEM. n.s. = not significant, *p < 0.05, **p < 0.01, ***p < 0.001 vs Control. Scale bars = 100 μm.

Because tumor angiogenesis [32] and lymphangio-genesis [33] are common features of VEGF-A, anti-angiogenic properties of the anticoagulants Fondaparinux and Tinzaparin were investigated. For *in vitro* angiogenesis studies, HUVECs were grown on Matrigel-covered surfaces in medium with 10% fetal calf serum and 1% growth supplement derived from bovine retina (containing growth factors) and saline control, Tinzaparin or Fondaparinux was added. Tube formation was quantified after 9 hours of incubation. Compared to saline control, a dose-dependent decrease in tube formation was detected in the presence of Tinzaparin (Figure 4A). By contrast, incubation with Fondaparinux did not block tubule formation beyond control levels, suggesting that Tinzaparin abolished angiogenesis by binding and blocking growth factors such as VEGF-A (Figure 4A). When the effects of the heparins were analyzed in tumor tissues *in vivo* by CD31 immunofluorescence stainings, Tinzaparin strongly and significantly reduced the intratumoral blood vessel density and size, as compared with control tumors, whereas the anti-angiogenic effect of Fondaparinux was marginal (Figures 4B-4D). To study the role of luminal VWF fibers on angiogenesis, tumors obtained from ADAMTS13-deficient mice were analyzed. Notably, ADAMTS13 deficiency resulted in a prolonged lifetime for VWF fibers associated with higher vessel density and size, thus supporting a role of EC activation and luminal VWF networks in tumor angiogenesis (Figures 4B-4D). Figure 4E, which plots the blood vessel density against the percentage of vessels with VWF fibers, shows a strong correlation among EC activation, VWF fiber formation and angiogenesis.

Moreover, the effects of the clinically approved anticoagulants on the intra- and peritumoral lymphatic vessel density in primary melanoma were examined in tissue sections of the primary tumors by Lyve-1 immunofluorescence stainings. To analyze the peritumoral lymphatic vessel density, the number of lymphatic vessels that crossed a 1-mm long line perpendicular to the primary tumor margin was determined. Neither Tinzaparin nor Fondaparinux showed any activity on lymphangiogenesis (Figure 4F). Taken together, our results suggest that tumor-mediated angiogenesis but not lymphangiogenesis is blocked by Tinzaparin. Moreover, tumor angiogenesis directly correlates with intraluminal VWF fiber formation.

### Tinzaparin and Fondaparinux treatments reduce primary tumor growth

**Figure 5:**
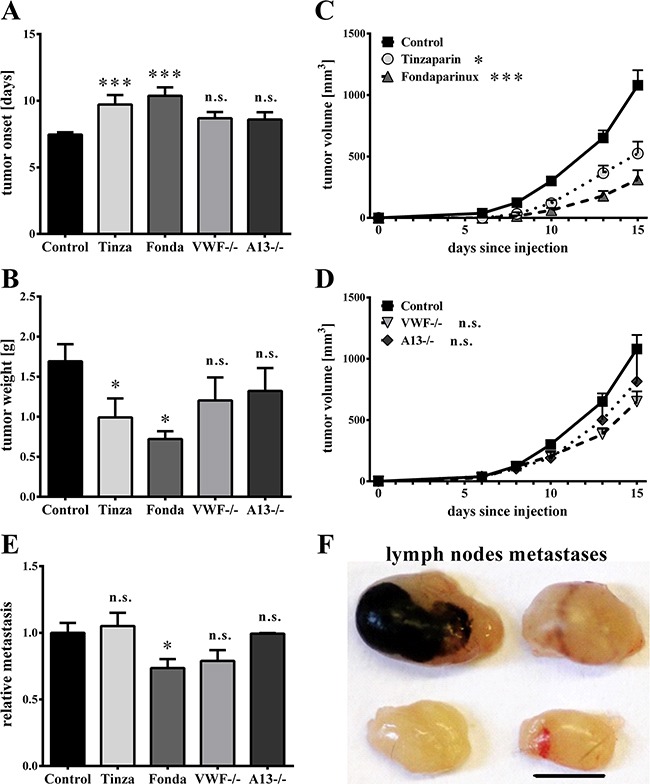
Luminal von Willebrand factor fibers have no effects on primary tumor growth or lymphatic metastasis After intradermal injections of Ret tumor cells into wild type, VWF−/− or ADAMTS13(A13)−/− mice, primary tumor growth was measured daily with a digital caliper. Animals were treated with Tinzaparin (Tinza), Fondaparinux (Fonda) or a saline control. **(A)** Both Tinzaparin (Tinza) and Fondaparinux (Fonda) delay the development of visible tumors. **(B)** Tumor weight and **(C+D)** tumor growth are affected by Tinzaparin and Fondaparinux but not by deficiencies in either VWF or ADAMTS13. **(E+F)** To analyze lymphatic metastasis formation of melanoma cells, inguinal and axial lymph nodes were dissected 15 days after tumor cell injections and metastases were densitometrically analyzed. **(E)** Whereas Tinzaparin does not affect metastasis formation in lymph nodes, quantification shows a slight reduction after Fondaparinux treatment. There were no significant differences in metastatic foci within the lymph nodes of VWF−/− and A13−/− animals compared to control mice. **(F)** Representative pictures of inguinal (upper row) and axial (lower row) lymph nodes. N = 6 – 14 mice per group. Data are presented as the mean ± SEM. n.s. = not significant, *p < 0.5, ***p < 0.001 vs Control. Scale bar = 5 mm.

Because earlier studies have demonstrated an impact of thrombin [34] and VEGF-A [32] on tumor progression, the effects of Fondaparinux and Tinzaparin on tumor growth were tested. Melanoma cells were intradermally injected into mice to establish a mouse model characterized by a primary tumor and lymph node metastasis. Mice received daily subcutaneous heparin treatments for 14 days. Both, Tinzaparin and Fondarparinux significantly delayed the development of macroscopically visible tumors by approximately 2.5 days, as compared to the vehicle-treated controls (Figure 5A). Moreover, the reduction in tumor growth was reflected by a reduced tumor weight of 1.0 g ± 0.2 g for Tinzaparin and 0.7 g ± 0.1 g for Fondaparinux compared with the vehicle-treated control (1.7 g ± 0.2 g; Figure 5B) and a reduced tumor volume (Figure 5C). To evaluate the role of VWF fibers on primary tumor growth, we analyzed tumor growth in wild type (WT) mice, VWF−/− and ADAMTS13−/− mice. There were no significant differences in tumor development (Figure 5A+5B+5D), thus excluding VWF fibers as critical determinants for primary tumor growth. This result was further confirmed by plotting the tumor weight as a function of the vessels with VWF fibers, which showed no significant correlation (Supplementary Figure S5). In summary, both anticoagulants inhibited tumor development to a similar extent, thus suggesting a greater role for thrombin in primary tumor growth.

### VWF fiber generation has no effects on lymph node metastasis

Before forming metastases in distal organs, early-stage malignant melanoma metastasizes into sentinel lymph nodes [35]. To study the role of VWF in lymphatic tumor dissemination, intradermal injections of Ret melanoma cells were performed, resulting in primary tumor development within the skin and in metastatic tumors in the lymph nodes. Melanin-containing tumor cell colonies in the lymph nodes were densitometrically quantified (Figure 5E+5F). To investigate whether inhibition of EC activation with subsequent VWF fiber release can abolish tumor progression through the lymphatics, heparin treatments were analyzed. Fondaparinux significantly reduced lymph node metastasis formation by 26.4%, whereas Tinzaparin treatment did not affect the lymphatic spread of melanoma cells (Figures 5E). Notably, wild type, VWF- and ADAMTS13-deficient mice developed lymph node metastases to similar extents, showing that VWF fibers are not required for lymphatic metastasis (Figures 5E). In summary, the results indicate that EC activation and VWF fibers within the tumor vasculature have no significant effects on lymphatic tumor spreading.

### Primary tumors orchestrate endothelial cell activation in distal organs

**Figure 6:**
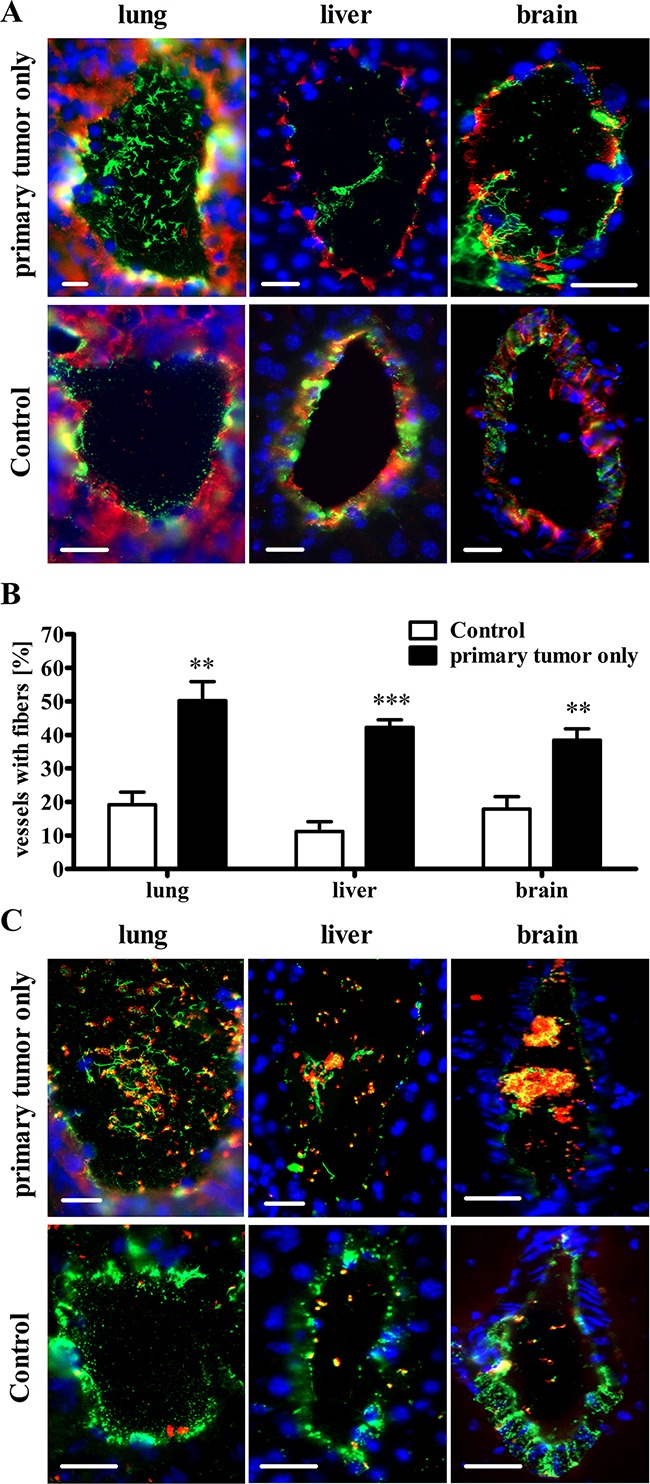
The primary tumor mediates activation of the vascular endothelium and VWF fiber formation in the microvasculature of distal organs without metastases Mice were sacrificed 15 days after intradermal inoculations of tumor cells. Endothelial cell activation in vessels of lung, liver and brain was determined relative to healthy wild type organs. **(A+B)** Cryosections from healthy WT and tumor-bearing WT mice were stained for VWF (green) and the endothelial marker CD31 (red). Nuclei were stained with DAPI (blue). **(A)** Representative images of healthy control organs show that VWF is mainly stored in the blood vessel walls of lung, liver and brain tissue. However, VWF fibers are detectable within the vessel lumina of the distal organ-specific vessels of mice bearing a primary tumor. **(B)** Quantification shows an increased number of vessels with luminal VWF fiber formation in organs from primary tumor-bearing mice compared with healthy tumor-free control animals. **(C)** Immunofluorescence stainings for VWF (green) and the platelet marker GPIb (red) show that VWF fibers mediate platelet binding and thrombus formation in the vessels of tumor-primed organs without metastases. Blood vessels (n = 49 – 351) from 4-7 animals per group were analyzed. Data are presented as the mean ± SEM. **p < 0.01, ***p < 0.001 vs Control. Scale bars = 20 μm.

We hypothesized that cancer-related VTE is a consequence of VWF-mediated platelet aggregation in organs distal to the primary tumor. Therefore, we analyzed blood vessels from lung, liver and brain tissues by immunofluorescence 15 days after intradermal injection of Ret cells. This time-point represents a transient stage in tumor development, at which metastases were undetectable in the indicated organs. We identified numerous vessels with VWF networks in the lumina of all analyzed organs (Figure 6A+6B) mediating platelet binding and aggregation (Figure 6C). The lungs were especially prone to thrombotic vessel occlusion. At 50.2 ± 5.8%, the percentage of vessels with fibers in the lung tissue was twice as high as that in the primary tumor (23.2 ± 3.1%). In the healthy lungs of tumor-free mice, 19.1 ± 3.8% of the vessels exhibited intraluminal VWF networks. The percentages in the liver and brain tissues from animals with primary tumors were 42.2 ± 2.5% and 38.4 ± 3.5%, respectively, whereas the percentages in the healthy control liver and brain tissues were 11.2 ± 2.9% and 17.9 ± 3.6%, respectively (Figure 6B; Supplementary Table S5). Importantly, the VWF fiber density in the vasculature of primary tumors was twice as high as that in the brain vessels (Supplementary Figure S6). These results strongly suggest that the primary tumor orchestrates EC activation followed by intraluminal VWF fiber generation in distal organs during a limited tumor stage (primary tumor without distal organ metastases). In addition to promoting tumor-associated thrombosis, this phenomenon may reflect melanoma recruitment in early stages of metastasis formation.

### Intraluminal VWF fibers promote hematogenous metastasis

To investigate the role of EC activation and VWF formation in hematogenous tumor dissemination, we used a second mouse model based on the induction of lung metastases by intravenous (i.v.) injection of Ret melanoma cells. The animals were sacrificed 14 days after the tumor cell injections, and the lungs were macroscopically analyzed for metastases and by immunofluorescence microscopy for examination of EC activation.

**Figure 7:**
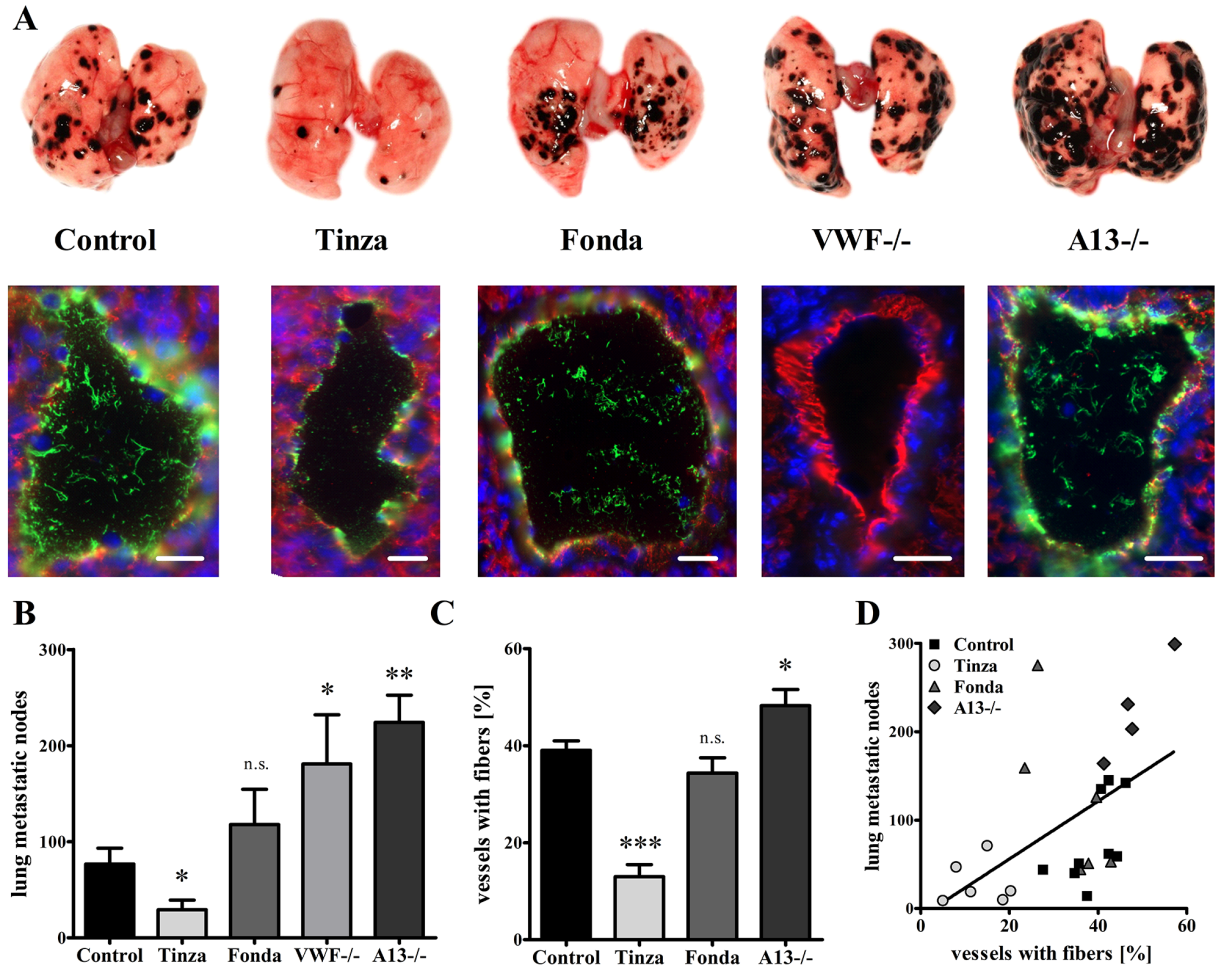
Endothelial cell activation and intraluminal VWF fibers mediate lung metastasis formation Ret melanoma cells were injected into the tail vein of wild type, VWF−/− and ADAMTS13 (A13−/−) mice. Animals were treated with Tinzaparin (Tinza), Fondaparinux (Fonda) or saline control for 15 days. Mice were sacrificed, and metastatic nodes in the lungs were macroscopically quantified. VWF formation in lung vessels was analyzed by immunofluorescence microscopy for VWF (green) and CD31 (red) using cryosections. **(A+B)** Animals treated with Tinzaparin (Tinza) show a strong decrease in lung metastasis compared with the saline treated controls, whereas Fondaparinux (Fonda) shows no therapeutic effects. However, deficiencies in VWF or ADAMTS13 result in a strong increase in lung metastases. **(A+C+D)** The immunofluorescence analysis and its corresponding quantification demonstrate a positive correlation between pulmonary VWF fiber formation and metastatic burden. Blood vessels (n = 235 – 549) from 4-9 animals per group were analyzed. Data are presented as the mean ± SEM. n.s. = not significant, *p < 0.05, **p < 0.01, ***p<0.001 vs Control. Scale bar = 20 μm.

In the lungs from Tinzaparin-treated animals the number of metastases (29.3 ± 10.1) was significantly reduced compared with vehicle-treated controls (76.9 ± 16.6 metastases) (Figure 7A+7B). However, no inhibition of metastasis formation (118.0 ± 36.8) was observed in the animals treated with Fondaparinux. Similarly to the results obtained from primary tumor blood vessels (Figure 3), immunofluorescence studies revealed a reduction in VWF fiber formation in the lung vessels after treatment with Tinzaparin, but not Fondaparinux (Figure 7A+7C; Supplementary Table S6). To delineate the role of VWF fibers in hematogenous metastasis, we examined lung tissue from VWF- and ADAMTS13-deficient mice. We observed 181.0 ± 51.3 metastases for the VWF−/− group, confirming previous studies by Terraube et al. [24]. Notably, we detected 224.3 ± 28.5 metastatic foci in lungs from ADAMTS13-deficient mice, which was approximately threefold higher than the number in the vehicle-treated control group (Figures 7A+7B). Interestingly, deficiencies in either VWF or ADAMTS13 resulted in significant increases in lung metastasis. A significant positive correlation between the number of metastases and VWF fiber generation in pulmonary vessels was observed across all groups (Figures 7D). Thus, these findings emphasize a pro-metastatic mechanism by which EC activation and VWF fiber formation promote hematogenous tumor dissemination.

## DISCUSSION

In this study, we report that the tumor microenvironment promotes EC activation, as reflected by intraluminal VWF fibers present in the tumor microvasculature and in tumor-free distal organs. We identified characteristics of VTE, including VWF-platelet aggregates and vessel occlusion, in multiple organs distal to the primary tumor that suggest a plausible mechanism for thrombosis in cancer patients. Moreover, VWF fibers promoted the formation of *in vivo* lung metastases in a mouse model for hematogenous tumor dissemination, but they exhibited no detectable effects on lymphatic metastasis. Inhibition of thrombin formation with Fondaparinux, a heparin anticoagulant, was not sufficient to block VWF network generation and metastasis. However, VEGF-A binding by the LMWH Tinzaparin with subsequent inhibition of luminal VWF fiber generation explained Tinzaparin's additional anti-metastatic potential and provides novel insights into the mechanisms underlying cancer-associated coagulation and metastasis.

VWF is stored in the ECs of the vessel wall and mediates platelet adhesion to the subendothelial matrix of damaged endothelium after vessel injury [36]. In addition to the crucial physiological effects of VWF in vessel repair, the release of VWF from intact, undamaged ECs can be induced by tumor cells [13–15]. The resulting luminal VWF fiber formation mediates the binding, activation and aggregation of circulating platelets (Supplementary Figure S2) [16, 18, 21]. This study describes an *in vitro* melanoma cell-induced VWF release (Figure 1) and an *in vivo* formation of luminal fibers in tumor microvessels (Figure 2). In agreement with the results of an *in vivo* canine microcirculation study that showed a correlation between increasing shear stress and decreasing in vessel diameters [37], detailed analyses of our data revealed higher VWF fiber densities in vessels with smaller diameters (Figure 2). Because the shear rates required for the unfolding and activation of VWF are approximately 200 s ^−1^, it is likely that VWF fiber formation is relevant in the microvasculature, with shear rates of 100 s ^−1^ to 8000 s ^−1^ [38]. The critical role of VWF in tumor progression is further supported by previous studies demonstrating that melanoma cells are able to stimulate undamaged ECs via tissue factor and subsequent thrombin generation, thereby stimulating the endothelial protease activator receptor-1 (PAR-1) [14]. Thrombin activates platelets, which are critical mediators of tumor cell implantation and serve as bridges to the endothelial surface through the highly adhesive VWF [10, 34]. PAR-1 has been proposed as a target for impeding the progression of malignant melanoma [39]. However, PAR-1 deficiency, which attenuates endothelial thrombin signaling, has been found not to affect metastasis formation in a tumor mouse model [25], thus suggesting that thrombin is not the only mechanism by which tumor cells exert their stimulatory activities. In this study, the anticoagulant LMWH Tinzaparin and the pentasaccharide Fondaparinux were used to explore the underlying mechanisms. Tinzaparin is known to inhibit thrombin generation and VEGF-A-mediated cell stimulation [21, 40, 41]. In contrast, Fondaparinux inhibits factor Xa and therefore thrombin generation but lacks an affinity for VEGF-A [21, 42]. Notably, Fondaparinux did not block EC activation *in vitro* in a system that lacked the clotting factors required for thrombin generation. However, the LMWH Tinzaparin with a higher molecular weight, inhibited tumor cell-secreted VEGF-A and abolished VWF release to the same extent as Bevacizumab (Figure 1). Moreover, the LMWH blocked luminal ULVWF release in the primary tumor microvasculature more effectively than Fondaparinux (Figure 3). This result suggests that VEGF-A is an additional mediator of EC activation and VWF network formation in the tumor vasculature, thus indicating a functional redundancy between thrombin and VEGF-A.

In addition to inhibiting EC activation, heparins affect angiogenesis [41]. LMWHs suppress the formation of thrombin, which is highly involved in regulating angiogenic processes [39, 43]. They also block the generation of fibrin fibers, which provide scaffolds for new blood vessel formation [44]. The results of this study clearly demonstrate a reduction in thrombin activity and in the thrombin-induced fibrin networks of the tumor vasculature after treatment with Fondaparinux or Tinzaparin (Figure 3). However, unlike Fondaparinux, Tinzaparin inhibited EC tube formation *in vitro* (Figure 4). Because clotting factors were absent in our *in vitro* settings, angiogenesis was additionally quantified in primary melanoma tissue, thus confirming that the inhibition of angiogenesis was lower with Fondaparinux compared to Tinzaparin (Figure 4). VEGF-A inhibition indicated a second mechanism for the Tinzaparin-mediated suppression of angiogenic processes. Indeed, Fondaparinux exhibits a minimal inhibitory effect on angiogenesis, whereas Tinzaparin has stronger anti-angiogenic activities [45, 46]. Moreover, Dalteparin promotes VEGF-A-mediated angiogenesis *in vivo* [47]. We have recently demonstrated that Fondaparinux exhibits a low binding affinity for VEGF-A, whereas Tinzaparin binds the growth factor with a high affinity [21]. In this context, it was demonstrated that VWF-deficiency is associated *in vitro* with increased angiopoietin-2 release and VEGF receptor-2-dependent proliferation and migration of ECs [48]. This result was confirmed *in vivo* by showing an increased angiogenesis in VWF −/− mice. Tumors in ADAMTS13-deficient animals exhibited an increased number of vessels in this study, thus further supporting a role for VWF in regulating vascular processes (Figure 4). Importantly, EC-released VWF fibers markedly bind platelets (Figure 6) releasing platelet-derived angiogenic factors, such as VEGF-A, thrombin and platelet activating factor [49–51].

Our previous studies have shown that blocking luminal VWF fiber formation is associated with tumor weight reduction and survival benefits in a *ret* transgenic mouse model of malignant melanoma, providing evidence of a pro-metastatic role for VWF [21]. The limitation of the *ret* model is the heterogeneity in tumor development and progression, making analysis of the molecular mechanisms of cancer dissemination and the efficacy of therapeutic agents difficult [52, 53]. Because the initial spread of malignant melanoma into lymph nodes defines the tumor staging and the patient prognosis [54], orthotopic tumor models allow the delineation of metastatic mechanisms. In our lymphatic tumor model, neither VWF deficiency nor ADAMTS13 deficiency had a significant effect on primary tumor development and metastasis formation (Figure 5). Treatment with Fondaparinux or Tinzaparin exhibited inhibitory effects on tumor growth, but only Fondaparinux reduced lymph node metastases. These observations indicate a stronger effect of thrombin than VEGF-A on lymphatic metastasis formation, which is consistent with results from a report showing a growth inhibition of implanted tumors that is associated with thrombin inhibition [55].

Because rapid primary tumor growth in this mouse model limits the development of detectable metastases in organs [56] and because clinical studies have provided evidence of a higher risk for VTE in patients within a limited disease stage typified by a primary tumor and lymph node metastasis [57], we speculated that thrombotic vessel occlusion is predominantly observed in the vascular beds of organs distal to the primary tumor. Indeed, we detected a substantial increase in EC activation and luminal VWF fiber formation in metastasis-free livers, brains and lungs (Figure 6), which are the most frequent target organs for malignant melanoma metastasis [58]. Thus, this study emphasizes a direct role of EC activation and VWF in triggering thrombosis in cancer patients already in a limited tumor stage (lymph node metastases, but no hematogenous tumor spread).

Previous findings have raised the question of whether VWF plays a pro- or anti-metastatic role in hematogenous tumor progression. Studies using the VWF−/− mouse model have produced similar results to ours (Figure 7) by demonstrating an increase in metastatic foci in lung tissues [24, 59]. Others have observed reduced metastasis formation after treatment with anti-VWF antibodies [23]. The complete absence of VWF in the genetic mouse model is characterized by missing WPBs [60], and therefore a dysregulated secretion of pro-metastatic factors, such as P-selectin and angiopoietin-2 [48, 61]. This dramatic alteration of EC physiology may be responsible for an increase in lung metastases in VWF-deficient animals. However, an incomplete, antibody-mediated inhibition of the multimeric glycoprotein VWF by targeting binding sites for platelets, ADAMTS13, heparin, collagen and other ligands [38] may account for the observed reduction in metastatic foci. Here, we used ADAMTS13-deficient mice, characterized by prolonged VWF fiber lifetimes, to analyze the role of VWF in metastasis formation. Interestingly, the mice showed a strong increase in lung metastases (Figure 7), thus confirming a unique role of intraluminal VWF fibers in the extravasation of melanoma cells. Furthermore, in contrast to Fondaparinux treatment, therapeutic administration of Tinzaparin exhibited a strong inhibition of EC activation in lung vessels and anti-metastatic effects (Figure 7). Therefore, inhibition of VEGF-A is a non-anticoagulant mechanism by which Tinzaparin exerts its anti-metastatic activities.

To date, the anti-cancer properties of heparins have been attributed to the attenuation of angiogenesis, galectin-3, heparanase and the inhibition of selectins (P- and E-selectin) [29, 62]. Although numerous studies, together with our data, have envisioned LMWHs as the treatment of choice for metastasis and cancer-related VTE, insights from clinical trials are conflicting [63]. Clinical observations using Dalteparin (CLOT trial) and Tinzaparin (CATCH trial) have demonstrated the advantages of LMWHs versus oral anticoagulants for the treatment of cancer-associated VTE [64, 65]. Other clinical studies such as the FAMOUS trial have shown only a moderate survival benefit associated with Dalteparin treatment in a subpopulation of cancer patients [66] and in patients with solid tumors without metastatic disease [67]. Furthermore, a recent trial targeting pancreatic cancer patients with Enoxaparin has reported a lack of anti-cancer effects for the LMWH [68]. However, the first clinical observation demonstrating a significant survival benefit in tumor patients used Logiparin, which subsequently became known as Tinzaparin [69]. The conflicting trial results indicate that detailed investigations will be necessary to assess the therapeutic limitations of LMWHs. The clinical investigations were performed during distinct disease progression stages, with different tumor entities and different anticoagulant heparins. Patients in the early stages of malignancy may have better responses to LMWH treatments. The therapeutic efficacy may have been underestimated, owing to the inclusion of diverse tumor types in these clinical trials, thus suggesting tumor type-specific mechanisms of tumor progression. Additionally, all clinically approved LMWHs are known to inhibit coagulation. However, because LMWHs have wide-ranging non-anticoagulant activities that are size-, molecular weight- and sulfation-dependent, a detailed characterization of their anti-metastatic potential is needed.

In conclusion, these data describe a plausible mechanism underlying the role of VWF fibers in cancer-related VTE and metastasis. Moreover, our work envisions the tumor cell-endothelial cell axis as a potential therapeutic target for malignancy and highlights the urgent need for an experimental delineation of the non-anticoagulant activities of LMWHs required for the design of innovative anti-metastatic strategies.

## MATERIALS AND METHODS

### Cell culture

The murine melanoma cell lines (Ret and B16F10) were maintained in RPMI supplemented with 10% heat-inactivated fetal calf serum (FCS), 1% non-essential amino acids, 1% glutamic acid and 1% penicillin/streptomycin. Human umbilical vein endothelial cells (HUVECs) were obtained and maintained as previously described [14]. Lymphatic endothelial cells (LECs) were purchased from Lonza (Switzerland) and maintained in EBM-2 supplemented with EGM-2 MV Single Quots. All cells were cultured at 37°C and 5% CO_2_.

### Mouse models

All animal experiments were approved by the governmental animal care authorities. C57BL/6J (wild type, WT) mice, VWF-deficient mice (VWF−/−, C57BL/6J-background) and ADAMTS13-deficient mice (ADAMTS13−/−, C57BL/6J-background) were purchased from The Jackson Laboratory (USA) and obtained from in-house breeding. All mice were maintained under specific-pathogen-free conditions. To study the effects of VWF fibers on EC activation and metastasis formation, we injected melanoma cells into 8 – 12 week-old female mice by using two distinct techniques: intradermal injection and intravenous injection. For intradermal injections, confluent melanoma cells (Ret or B16F10) were trypsinized, washed and centrifuged twice and diluted in PBS to 750,000 cells per 100 μl. Each 100-μl cell suspension was intradermally injected into the shaved dorsal skin of each mouse. Primary tumor growth was assessed with a digital caliper with the following formula: Tumor volume = ½(length × width^2^). After 15 days, mice were sacrificed to assess tumor weight with a laboratory scale. Lymph nodes were dissected and photographed beside a standardized gray scale with a Canon EOS 60D reflex camera and a Tamron AF 90 mm f/2.8 macro lens. Because metastasis of malignant melanoma is associated with lymph node blackening, the gray scale value of each lymph node was compared with a reference gray scale to quantify lymphatic metastasis. For the intravenous model, we intravenously injected 120,000 Ret cells into the tail veins of anesthetized mice to induce lung metastases. The mice were sacrificed after 14 days, and the metastatic foci on the lung surfaces were counted by two independent investigators. For anticoagulant treatments, we subcutaneously injected mice with Tinzaparin (600 IE/kg), Fondaparinux (150 μg/kg) or a saline control starting one day before the inoculation of melanoma cells. After the mice were sacrificed, the excised tumors and organs were embedded into Tissue-Tek and snap frozen at -80°C for cryosectioning.

### Generation of melanoma-derived supernatants

To generate supernatants, 5 × 10^5^ melanoma cells were grown to confluence in T75 flasks for 40 hours. After aspiration of the culture medium, the cells were rinsed twice with HEPES-buffered Ringer's solution (HBRS: 140 mM NaCl, 5 mM KCl, 1 mM MgCl_2_, 1 mM CaCl_2_, 5 mM glucose and 10 mM HEPES) and incubated in 5 ml of HBRS alone or with Tinzaparin (100 U/ml) or Fondaparinux (50 μg/ml) at 37°C and 5% CO_2_. After an incubation of 8 hours, the supernatants were collected, centrifuged and stored at -80°C.

### Endothelial cell stimulation

Endothelial cells were grown to confluence in gelatin-covered 12-well plates. After being rinsed twice with HBRS, ECs were stimulated with VEGF-A (3 ng/ml), melanoma-derived supernatant alone or melanoma-derived supernatant containing Tinzaparin (100 U/ml), Fondaparinux (50 μg/ml) or Bevacizumab (0.65 mg/mL), the VEGF-A inhibitor. Thrombin (0.5 U/mL) was used as positive control, and HBRS served as negative control. After 15 minutes, EC supernatant was harvested, centrifuged and stored at -20°C for subsequent ELISA experiments. ECs cultivated on coverslips were used for immunofluorescence studies. ECs were exposed to orbital shear flow to allow the unrolling of released VWF using an orbital shaker (210 rpm; IKA, Germany). To evaluate VWF expression levels, confluent HUVECs were stimulated with a 1:1 mixture of nutrient-deficient medium and HBRS with indicated ingredients. After an incubation of 24 hours, the cells were immediately prepared for total RNA extraction.

### Immunofluorescence staining

The indirect immunofluorescence staining technique was used to visualize the morphological structures of cultivated endothelial cells and cryosections. The following primary antibodies were used at indicated concentrations: rabbit anti-human VWF (1:150; Dako, Denmark), rat anti-mouse CD31 (1:50; BD Biosciences, Germany), sheep anti-human fibrinogen (1:150, AbD Serotec, UK), rat anti-mouse Lyve-1 (1:100; BD Biosciences, Germany), rat anti-mouse GPIb (1:100; EMFRET Analytics, Germany), mouse anti-human CD31 (1:50; Dako, Denmark) and mouse anti-human VEGFR-3 (1:50; R&D Systems, USA). CD31 was used as a marker for vascular ECs, and Lyve-1 and VEGFR-3 were used for staining lymphatic ECs.

The following secondary antibodies were used: FITC-conjugated goat anti-rabbit (1:200; BD Pharmingen, USA), FITC-conjugated goat anti-rat (1:200; BD Pharmingen, USA), Alexa555-conjugated goat anti-rat (1:200; Invitrogen, USA), Alexa555-conjugated goat anti-mouse (1:200; Invitrogen, USA) and Alexa555-conjugated donkey anti-sheep (1:200, Invitrogen, USA).

For *in vitro* immunofluorescence staining of endothelial cells, HUVECs and LECs were seeded onto gelatin-coated coverslips and stimulated as described above. After stimulation, ECs were immediately fixed in ice-cold methanol for 30 min, rinsed twice and blocked with HBRS containing 2% bovine serum albumin for 45 minutes.

Tissue cryosections (10 μm) were fixed in ice-cold methanol for 30 min, washed in phosphate buffered saline containing 0.1% Tween 20 (PBS-T) and blocked in blocking solution (PBS-T containing 10% goat serum) for 45 minutes. The primary antibodies were diluted in PBS-T and incubated for 90 minutes. After cells were washed twice with PBS-T, the secondary antibodies were diluted 1:200 in the blocking solution and were applied for 50 minutes. Nuclei were counterstained with 4,6-diamidino-2-phenylindole (DAPI) for 10 min. Coverslips were mounted with DABCO/Mowiol. Immunofluorescence microscopy was performed with a Zeiss Axiovert 200 microscope. Images were processed with Carl Zeiss Imaging software, AxioVision software (4.8) and ImageJ (1.47c).

### Microscopic analysis of Immunofluorescence stainings

The analysis of immunofluorescence stainings was performed by a blinded, independent investigator. To determine the percentage of blood vessels containing luminal VWF or fibrin fibers, a sample was examined by microscopy if a vessel contained a visible lumen. If at least one VWF/fibrin fiber was present in the vessel lumen, the vessel was deemed positive for VWF/fibrin fibers. A fiber was defined as having minimum length of 5 μm. In cases of ambiguity, the vessel was photographed, and the length of the fiber was measured with AxioVision software. The percentage of blood vessels with VWF or fibrin fibers was assessed for each sample, and the average percentage was calculated for each group.

To analyze the VWF fiber densities, images were taken from all vessels containing visible lumina in each sample. Afterward, the diameter and cross sectional area of each vessel was measured with AxioVision software, and the corresponding number of VWF fibers was counted by an independent investigator. VWF fiber density in blood vessels was defined as the number of VWF fibers per 1,000 μm^2^ of the vessel cross sectional area.

For quantification of vascular and lymphatic vessel densities, overview pictures with standardized exposure times were acquired with AxioVision software. The vascular and intratumoral lymphatic vessel density was determined by measuring the cross sectional area of the tumor and the total area of the CD31- and Lyve-1-positive structures inside the tumor with ImageJ. The average vascular vessel size per tumor was defined as the average cross sectional area of the CD31-postive structures measured by the particle analyzer function of ImageJ. To determine the peritumoral lymphatic vessel density, the number of lymphatic vessels that crossed a 1-mm long line perpendicular to the primary tumor margin was determined.

### Enzyme-linked immunosorbent assay

The release of endothelial VWF release into the supernatants of Ret cells was quantified by the enzyme-linked immunosorbent assay technique, as previously described [14]. P-Selectin and angiopoietin-2 ELISAs (R&D Systems, USA) were performed according to the manufacturer's instructions.

### Thrombin generation

Mice received daily subcutaneous injections with Tinzaparin (600 IE/kg), Fondaparinux (150 μg/kg) or saline as control for 5 days. For the thrombin measurements, citrated whole blood was centrifuged for 15 minutes at 1000 x g, and an analysis of the plasma was performed according to the manufacturer's instructions (Technoclone GmbH, Austria).

### RNA preparation and qRT-PCR

Total ribonucleic acid (RNA) from HUVECs was isolated using the SV Total RNA Isolation System from Promega (USA) according to the manufacturer's protocol. The cDNA was synthesized from 1 μg of total RNA per sample using the Reverse Transcription System from Promega. To determine the mRNA transcript level from cDNA, quantitative real-time polymerase chain reaction (RT-qPCR) was performed using the GoTaq® 1-Step RT-qPCR System from Promega and specific primers to VWF or the β-actin housekeeping gene. VWF expression was normalized to β-actin mRNA expression for each sample.

### *In vitro* tube formation assay

For *in vitro* angiogenesis studies, the ibidi tube formation assay (ibidi, Germany) was performed according to the instructions of the manufacturer using ibidi μ-slides. Briefly, 200,000 HUVECs were suspended in 1 ml of M199 culture medium (Life Technologies, Karlsruhe, Germany) supplemented with 10% fetal calf serum, antibiotics (penicillin and streptomycin) and 1% growth supplement derived from bovine retina. Cell culture medium was supplemented with sodium, Tinzaparin or Fondaparinux at indicated concentrations. The cell suspension (50μl) was applied to the Matrigel-coated (BD Biosciences, USA) well of each μ-slide and incubated in a humidified chamber at 37°C. Tube formation was imaged at a 10-fold magnification at 0, 0.5, 3, 6 and 9 hours after cell seeding. The total tube length was quantified after 9 hours with ImageJ software.

### Statistical analysis

Values are expressed as mean ± SEM. Statistical analysis was performed with GraphPad Prism software to apply the unpaired student's t-test. P < 0.05 was considered to be statistically significant.

## SUPPLEMENTARY MATERIALS FIGURES AND TABLES


