# Supplementary material for: Heparins that block VEGF-A-mediated von Willebrand factor fiber generation are potent inhibitors of hematogenous but not lymphatic metastasis
Source: Oncotarget. 2016 Sep 2;7(42):68527–45. doi: 10.18632/oncotarget.11832 (PMC5356571; doi:10.18632/oncotarget.11832)
Supplement: Supplementary file 1 [file oncotarget-07-68527-s001.pdf]

Heparins that block VEGF-A-mediated von Willebrand factor fiber generation are potent inhibitors of hematogenous but not lymphatic metastasis

SUPPLEMENTARY FIGURES AND TABLES

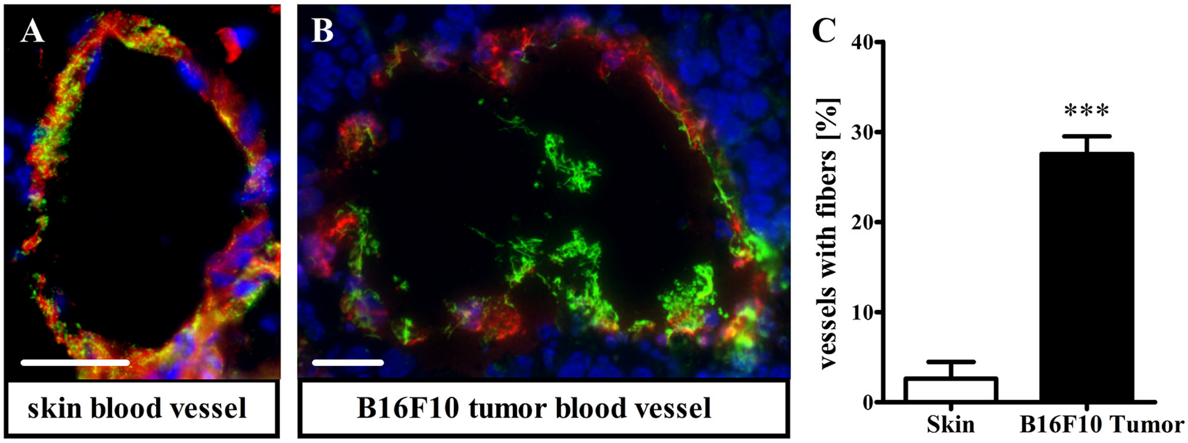

Supplementary Figure S1: Intraluminal VWF fiber formation occurs in blood vessels of intradermal B16F10 tumors. Tissue cryosections were stained for VWF (green) and CD31 (red). Nuclei were counterstained with DAPI (blue). **A.** In healthy skin vessels, VWF is stored in the endothelial cells of the vessel wall, indicating a quiescent endothelium. **B.** Tumor vessels show intraluminal VWF fiber formation and reduced endothelial VWF in the blood vessel wall, indicating endothelial cell activation. **C.** Quantification reveals increased VWF fiber formation in the B16F10 primary tumor vasculature. Data are presented as the mean ± SEM. Blood vessels (n = 76 – 519) from 4-5 animals per group were analyzed. ***p < 0.001. Scale bar = 10 µm.

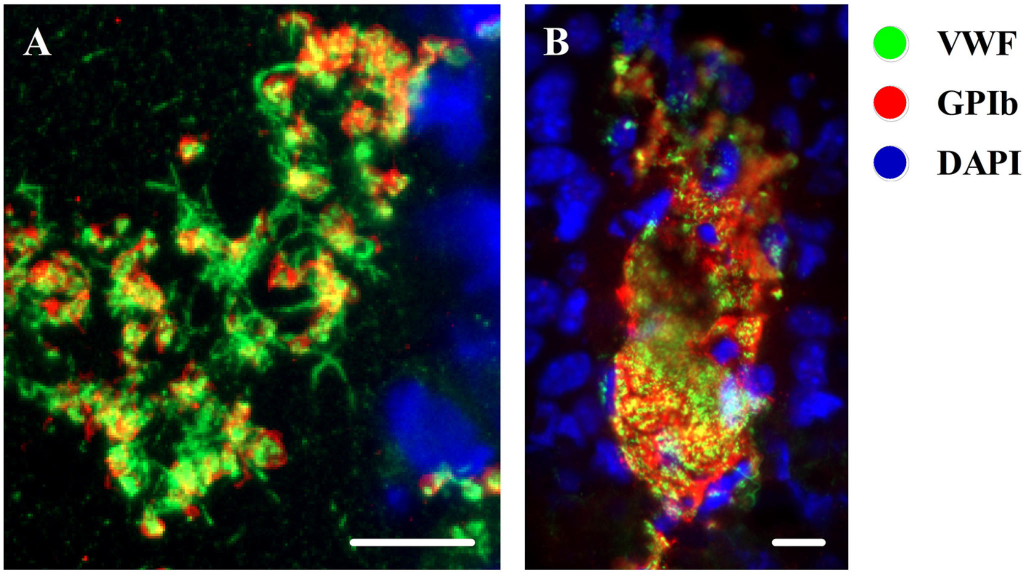

Supplementary Figure S2: Intraluminal VWF fibers bind platelets to induce thrombus formation. Cryosections of primary skin tumors were stained for VWF (green) and the platelet marker GPIb (red). Nuclei were counterstained with DAPI (blue). **A+B.** Representative images of tumor vessels demonstrate that VWF fibers within the lumen of a blood vessel mediate binding of platelets associated with thrombotic vessel occlusion. Scale bar = 10 µm.

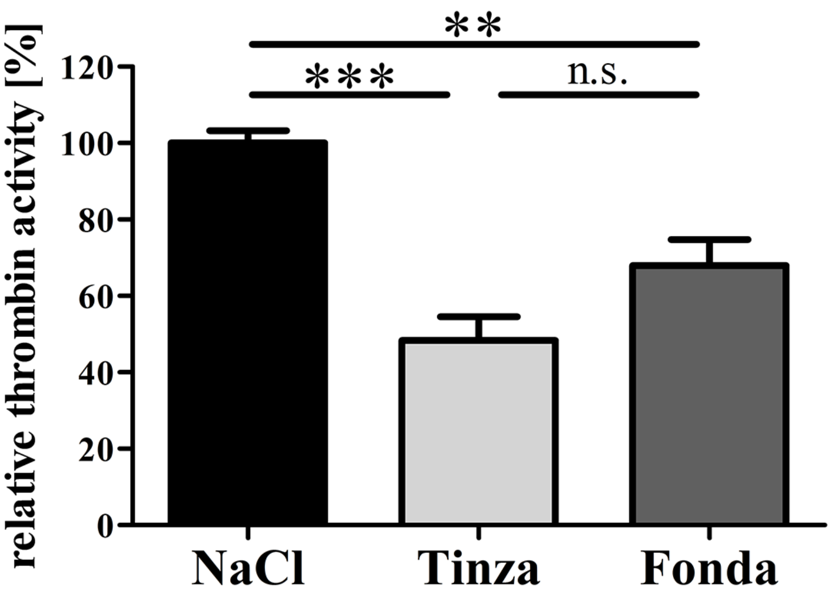

Supplementary Figure S3: Tinzaparin and Fondaparinux are potent inhibitors of thrombin. Thrombin activity was measured after subcutaneous injection of Tinzaparin, Fondaparinux or saline as control for 5 days in citrated plasma. Quantification reveals a significant decrease in thrombin activity after Tinzaparin (Tinza) and Fondaparinux (Fonda) treatment compared with the control (NaCl). The blood from 4-6 animals per group was analyzed. Data are presented as the mean ± SEM. n.s. = not significant, $^{**}p < 0.01$, $^{***}p < 0.001$.

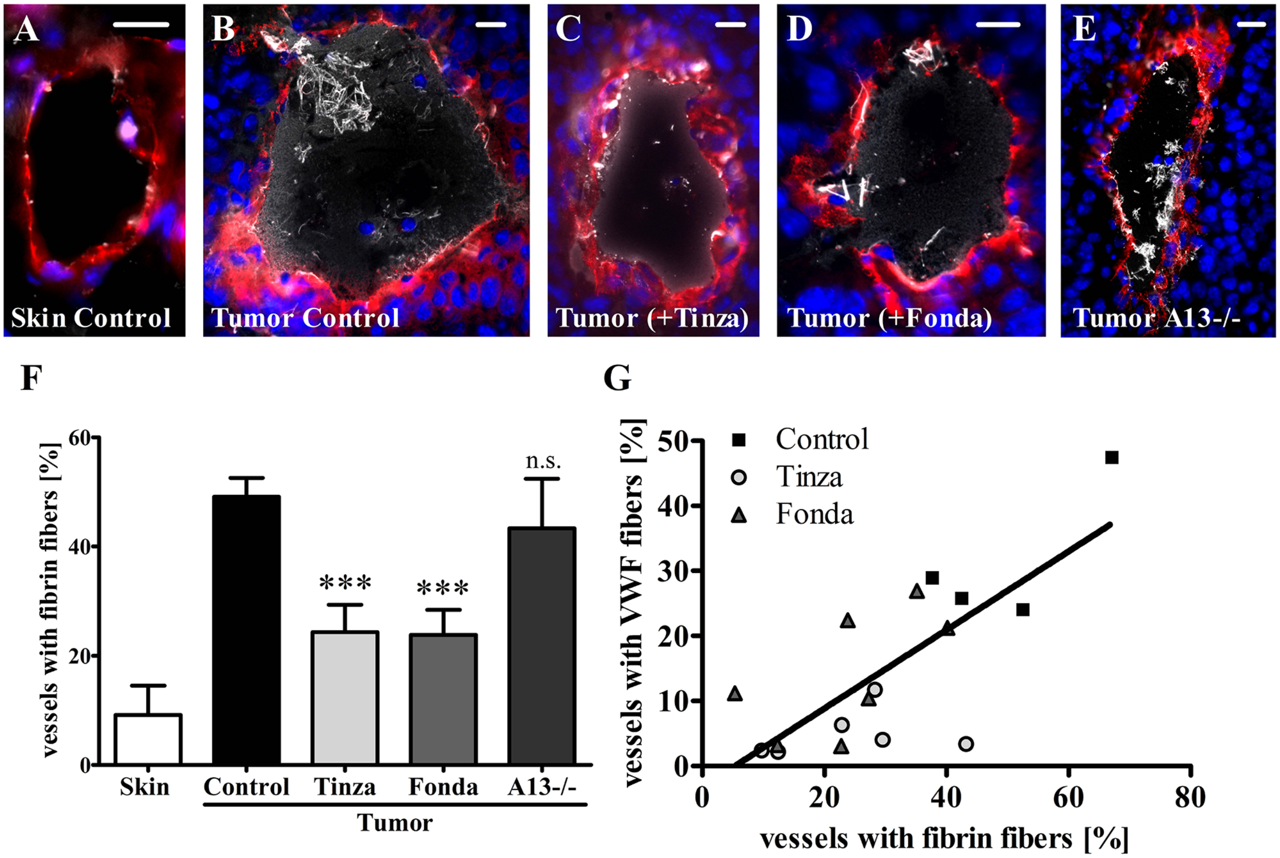

Supplementary Figure S4: VWF fiber generation correlates with fibrin fiber formation in tumor blood vessels. Immunofluorescence staining for fibrin (white) and CD31 (red) in cryosections of healthy skin or intradermally growing Ret tumors was performed. Nuclei were stained with DAPI (blue). **A – E.** Representative images of fibrin fiber formation in healthy skin **(A)**, a control tumor **(B)**, a tumor from a Tinzaparin (+Tinza)-treated mouse **(C)**, a tumor from a Fondaparinux (+Fonda)-treated mouse **(D)** and a tumor from an ADAMTS13 (A13)-/- mouse **(E)**. **F.** Quantification reveals a significantly decreased number of vessels with intraluminal fibrin fibers after the Tinzaparin and Fondaparinux treatments compared with the control. **G.** There is a positive correlation between VWF fibers and fibrin fiber formation ($r = 0.76$). Tumor vessels ($n = 516 – 857$) from 6-9 animals per group were analyzed and compared to healthy skin control ($n = 34$ vessels from 4 samples). Data are presented as the mean ± SEM. n.s. = not significant, ***$p < 0.001$ vs Tumor Control. Scale bars = 20 µm.

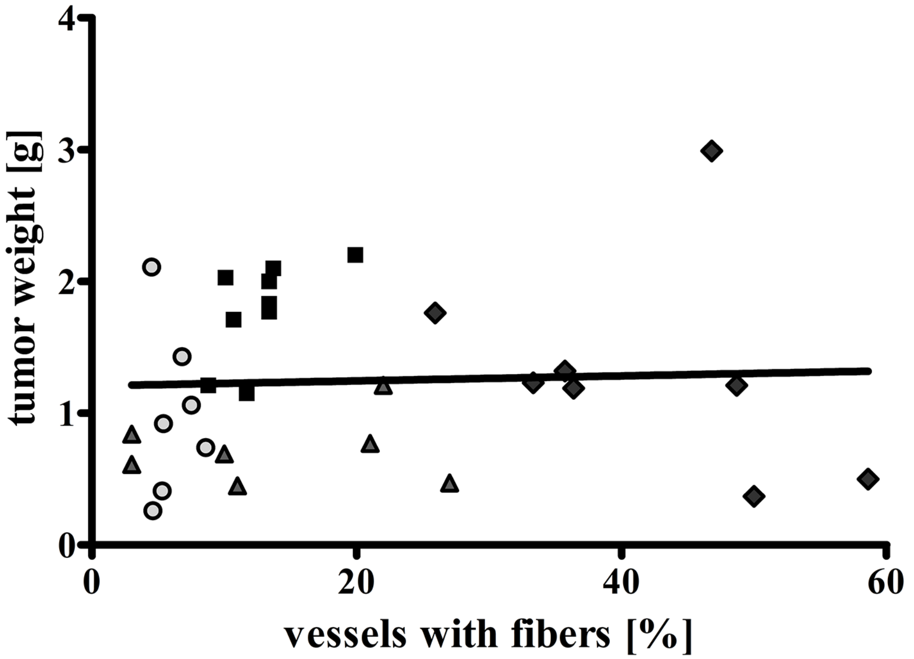

Supplementary Figure S5: Correlation of VWF fiber formation and tumor growth. Tumor weight as a function of the fiber-containing vessels shows no correlation between VWF-fiber formation and tumor growth (r = 0.25).

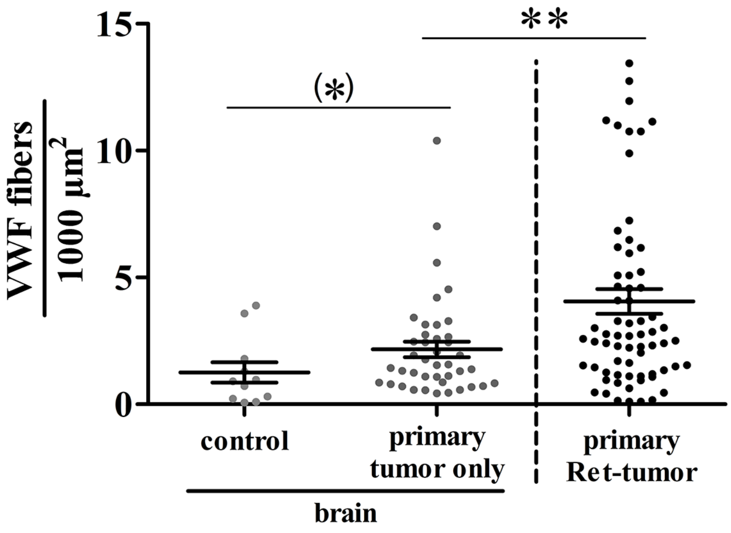

Supplementary Figure S6: The VWF fiber density in the tumor microvasculature is higher than that in brain vessels. Tissue sections of healthy brain tissue, of brain tissue from Ret tumor-bearing mice and primary tumor tissues were stained for VWF and CD31. Analysis of VWF fiber density expressed as the number of fibers per 1,000 µm^2 demonstrates a higher VWF density in activated brain vessels from tumor-bearing mice. The fiber density in the primary tumor is higher than in distal organ vessels. N = 4-6 animals per group. Data are presented as the mean ± SEM. n.s. = not significant, (*)p < 0.1, **p < 0.01.

Supplementary Table S1: Analysis of microvasculatures in healthy skin and primary Ret skin tumors

Group	Healthy skin		Ret Tumor	
Number of animals (n)	4		4	
Mean percentage of vessels with fibers/animal	2.0%		20.0%	
VWF fibers	-	+	-	+
Absolute number of vessels/group	74	2	244	69
Absolute percentage of vessels/group	97.4%	2.6%	88.0%	22.0%

Tissue cryosections were stained for VWF and CD31, and microvessels were analyzed for intraluminal VWF fiber formation. For the analysis, VWF fibers were defined as having a minimum length of 5 µm.

Supplementary Table S2: Analysis of microvasculatures in healthy skin and primary B16F10 skin tumors

Group	Healthy skin		B16F10 Tumor	
Number of animals (n)	4		5	
Mean percentage of vessels with fibers/animal	2.0%		25.9%	
VWF fibers	-	+	-	+
Absolute number of vessels/group	74	2	376	143
Absolute percentage of vessels/group	97.4%	2.6%	72.4%	27.6%

Tissue cryosections were stained for VWF and CD31, and microvessels were analyzed for intraluminal VWF fiber formation. For the analysis, VWF fibers were defined as having a minimum length of 5 µm.

Supplementary Table S3: Analysis of microvasculatures in healthy skin and primary Ret skin tumors

Group	Healthy skin		Tumor Control		Tumor (+Tinza)		Tumor (+Fonda)		Tumor A13-/-	
Number of animals (n)	4		9		6		7		6	
Mean percentage of vessels with fibers/ animal	9.2%		49.1%		24.3%		23.8%		43.3%	
Fibrin fibers	-	+	-	+	-	+	-	+	-	+
Absolute number of vessels/group	30	4	364	280	391	125	651	206	390	152
Absolute percentage of vessels/group	88.2%	11.8%	56.5%	43.5%	75.8%	24.2%	76.0%	24.0%	72.0%	28.0%

Mice were treated with Tinzaparin (+Tinza), Fondaparinux (+Fonda) or saline control. Tissue cryosections were stained for fibrin and CD31, and microvessels were analyzed for intraluminal fibrin fiber formation. For the analysis, fibrin fibers were defined as having a minimum length of 5 µm.

Supplementary Table S4: Analysis of microvasculatures in primary Ret skin tumors

Group	Tumor Control		Tumor (+Tinza)		Tumor (+Fonda)		Tumor A13-/-	
Number of animals (n)	14		7		7		7	
Mean percentage of vessels with fibers/animal	23.2%		4.9%		14.1%		38.3%	
VWF fibers	-	+	-	+	-	+	-	+
Absolute number of vessels/group	1621	593	1484	72	2052	317	966	714
Absolute percentage of vessels/group	73.2%	26.8%	95.4%	4.6%	86.6%	13.4%	57.5%	42.5%

Mice were treated with Tinzaparin (+Tinza), Fondaparinux (+Fonda) or saline control. Tissue cryosections were stained for VWF and CD31, and microvessels were analyzed for intraluminal VWF fiber formation. For the analysis, VWF fibers were defined as having a minimum length of 5 µm.

Supplementary Table S5: Analysis of microvasculatures in the lung, liver and brain tissues of healthy mice and mice with primary Ret skin tumors

Group	Lung				Liver				Brain			
	Control		Tumor		Control		Tumor		Control		Tumor	
Number of animals (n)	4		7		4		7		4		6	
Mean percentage of vessels with fibers/animal	19.1%		50.2%		11.2%		42.2%		17.9%		38.4%	
VWF fibers	-	+	-	+	-	+	-	+	-	+	-	+
Absolute number of vessels/group	137	34	109	115	308	43	192	151	41	8	75	48
Absolute percentage of vessels/group	80.1%	19.9%	48.7%	51.3%	87.8%	12.2%	56.0%	44.0%	83.7%	16.3%	61.0%	39.0%

Tissue cryosections were stained for VWF and CD31, and microvessels were analyzed for intraluminal VWF fiber formation. For the analysis, VWF fibers were defined as having a minimum length of 5 µm.

Supplementary Table S6: Analysis of microvasculatures in metastatic lungs

Group	Metastatic Lung Control		Metastatic Lung (+Tinza)		Metastatic Lung (+Fonda)		Metastatic Lung A13-/-	
Number of animals (n)	9		6		6		4	
Mean percentage of vessels with fibers/ animal	39.1%		13.0%		34.4%		48.3%	
VWF fibers	-	+	-	+	-	+	-	+
Absolute number of vessels/group	332	217	297	42	221	107	120	115
Absolute percentage of vessels/group	60.5%	39.5%	87.6%	12.4%	67.4%	32.6%	51.1%	48.9%

Ret cells were intravenously injected into the tail vein to induce lung metastasis formation. Mice were treated with Tinzaparin (+Tinza), Fondaparinux (+Fonda) or saline control. Tissue cryosections were stained for VWF and CD31, and microvessels were analyzed for intraluminal VWF fiber formation. For the analysis, VWF fibers were defined as having a minimum length of 5 µm.
